# Supplementary material for: Nutrients Intake in Individuals with Hypertension, Dyslipidemia, and Diabetes: An Italian Survey
Source: Nutrients. 2020 Mar 27;12(4):923. doi: 10.3390/nu12040923 (PMC7230242; doi:10.3390/nu12040923)

Table 1S. Characteristics of men and women receiving and not receiving treatment for hypertension. Men and women aged 35-79 years from Osservatorio Epidemiologico Cardiovascolare/Health Examination Survey of the CUORE Project 2008-2012.

	MEN									WOMEN								
	Not treated for hypertension				Treated for hypertension					Not treated for hypertension				Treated for hypertension				
	n	%	95% C.I.		n	%	95% C.I.		Sign.	n	%	95% C.I.		n	%	95% C.I.		Sign.
**Smoking Habit**	2672	25.8	24.2	27.5	1249	20.5	18.2	22.7	**	2770	21.9	20.4	23.5	1155	23.1	20.6	25.5	ns
**Phisical Inactivity**	2745	30.8	29.1	32.6	1280	37.3	34.7	40.0	**	2832	41.3	39.5	43.1	1171	45.1	42.3	48.0	ns
**Low Education**	2731	44.5	42.6	46.3	1268	49.3	46.5	52.0	*	2820	44.7	42.9	46.6	1147	60.4	57.6	63.3	***
**Overweight or Obesity**	2749	69.1	67.4	70.8	1280	85.9	84.0	87.8	***	2837	52.8	50.9	54.6	1174	76.6	74.2	79.0	***

Smoking Habit: smoking one or more cigarettes per day . Physical Inactivity: usually reading, watching television, going to the movies or spending leisure time in other sedentary activities. Low Education: primary or middle school. Overweight or Obesity: BMI >25.0 kg/m^2. C.I. = Confidence Interval. Sign: statistical significance of chi-squares to compare treated and not treated groups; *** p<0.0001; ** p<0.01; * p<=0.05; ns not statistically significant. Data are standardized by age using the 2013 European population.

Table 2S. Characteristics of men and women receiving and not receiving treatment for hypcholesteromia. Men and women aged 35-79 years from Osservatorio Epidemiologico Cardiovascolare/Health Examination Survey of the CUORE Project 2008-2012.

	MEN									WOMEN								
	Not treated for dyslipidemia				Treated for dyslipidemia					Not treated for dyslipidemia				Treated for dyslipidemia				
	n	%	95% C.I.		n	%	95% C.I.		Sign.	n	%	95% C.I.		n	%	95% C.I.		Sign.
**Smoking Habit**	3360	25.1	23.6	26.6	528	27.8	24.0	31.6	ns	3378	21.4	20.0	22.8	530	22.6	19.0	26.2	ns
**Phisical Inactivity**	3451	31.2	29.7	32.8	540	39.5	35.4	43.6	**	3452	42.8	41.1	44.4	536	51.8	47.6	56.1	**
**Low Education**	3435	44.0	42.4	45.7	533	49.8	45.5	54.0	ns	3424	46.9	45.2	48.5	526	51.2	47.0	55.5	ns
**Overweight or Obesity**	3455	72.1	70.6	73.6	540	77.8	74.3	81.3	ns	3456	56.2	54.6	57.9	537	67.8	63.8	71.8	***

Smoking Habit: smoking one or more cigarettes per day . Physical Inactivity: usually reading, watching television, going to the movies or spending leisure time in other sedentary activities. Low Education: primary or middle school. Overweight or Obesity: BMI >25.0 kg/m^2. C.I. = Confidence Interval. Sign: statistical significance of chi-squares to compare treated and not treated groups; *** p<0.0001; ** p<0.01; * p<=0.05; ns not statistically significant. Data are standardized by age using the 2013 European population.

Table 3S. Characteristics of men and women receiving and not receiving treatment for diabetes. Men and women aged 35-79 years from Osservatorio Epidemiologico Cardiovascolare/Health Examination Survey of the CUORE Project 2008-2012.

	MEN									WOMEN								
	Not treated for diabetes				Treated for diabetes					Not treated for diabetes				Treated for diabetes				
	n	%	95% C.I.		n	%	95% C.I.		Sign.	n	%	95% C.I.		n	%	95% C.I.		Sign.
**Smoking Habit**	3627	25.2	23.8	26.6	298	24.1	19.2	28.9	ns	3736	21.3	20.0	22.6	192	19.7	14.1	25.3	ns
**Phisical Inactivity**	3721	31.4	29.9	32.9	308	39.6	34.1	45.0	*	3813	42.1	40.5	43.6	193	55.8	48.8	62.8	**
**Low Education**	3699	43.7	42.1	45.2	305	64.0	58.7	69.4	***	3783	46.6	45.0	48.2	187	63.8	56.9	70.7	***
**Overweight or Obesity**	3725	72.0	70.6	73.4	308	83.3	79.1	87.4	**	3820	56.6	55.0	58.2	194	79.0	73.2	84.7	***

Smoking Habit: smoking one or more cigarettes per day . Physical Inactivity: usually reading, watching television, going to the movies or spending leisure time in other sedentary activities. Low Education: primary or middle school. Overweight or Obesity: BMI >25.0 kg/m^2. C.I. = Confidence Interval. Sign: statistical significance of chi-squares to compare treated and not treated groups; *** p<0.0001; ** p<0.01; * p<=0.05; ns not statistically significant. Data are standardized by age using the 2013 European population.

Figure 1S. Distribution of Total energy intake (Kcal/day) in men and women receiving and not receiving hypertension treatment. Men and women aged 35-79 years from Osservatorio Epidemiologico Cardiovascolare/Health Examination Survey of the CUORE Project 2008-2012.

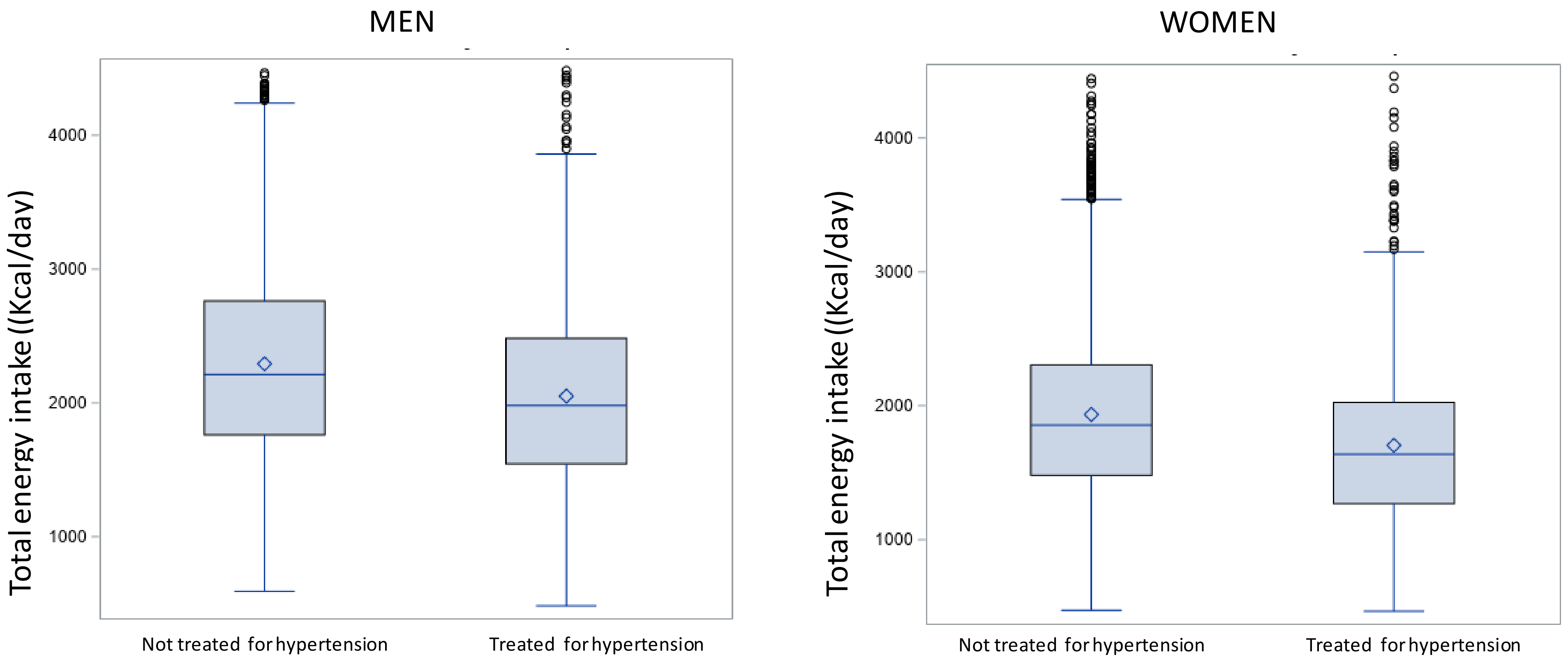

Figure 2S. Distribution of Total energy intake (Kcal/day) in men and women receiving and not receiving hypercholesterolemia treatment. Men and women aged 35-79 years from Osservatorio Epidemiologico Cardiovascolare/Health Examination Survey of the CUORE Project 2008-2012.

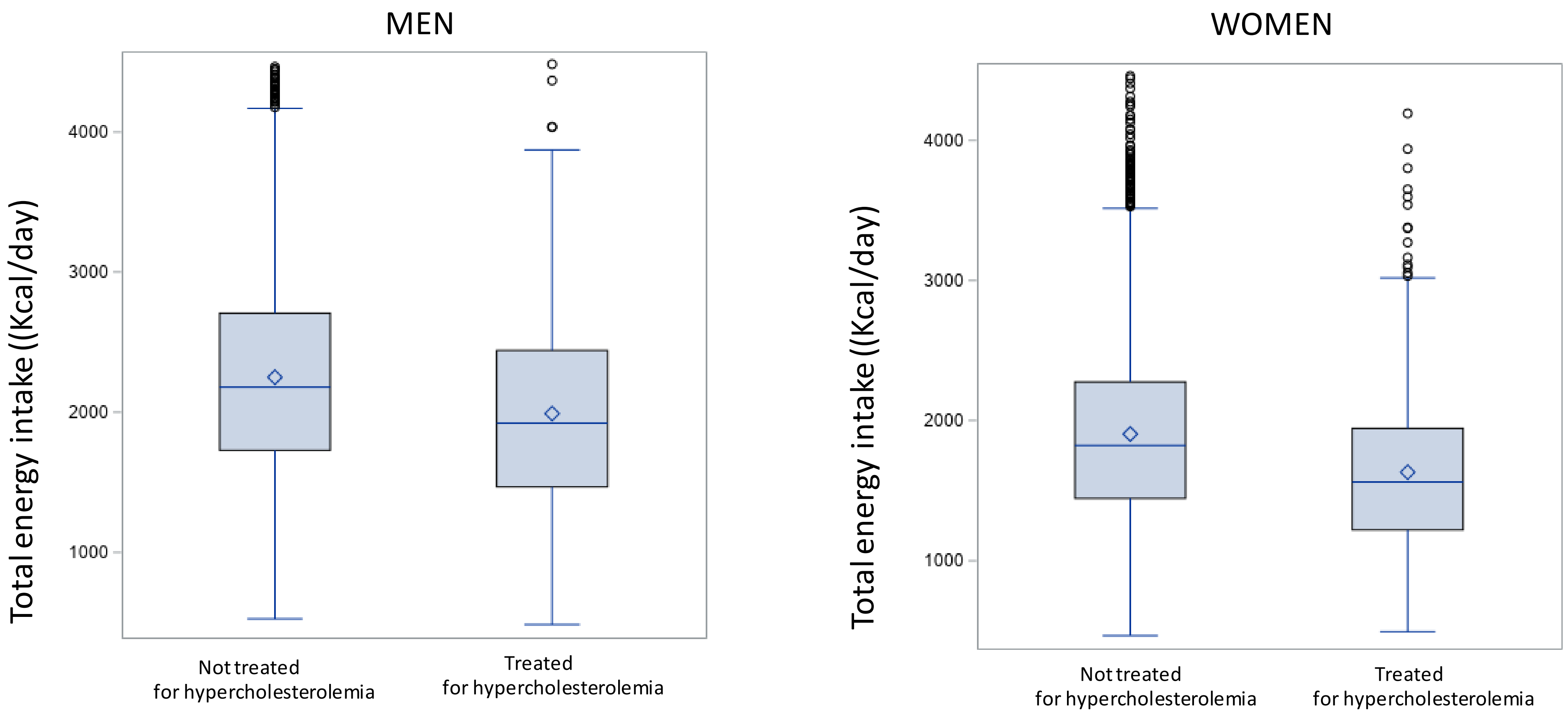

Figure 3S. Distribution of Total energy intake (Kcal/day) in men and women receiving and not receiving diabetes treatment. Men and women aged 35-79 years from Osservatorio Epidemiologico Cardiovascolare/Health Examination Survey of the CUORE Project 2008-2012.

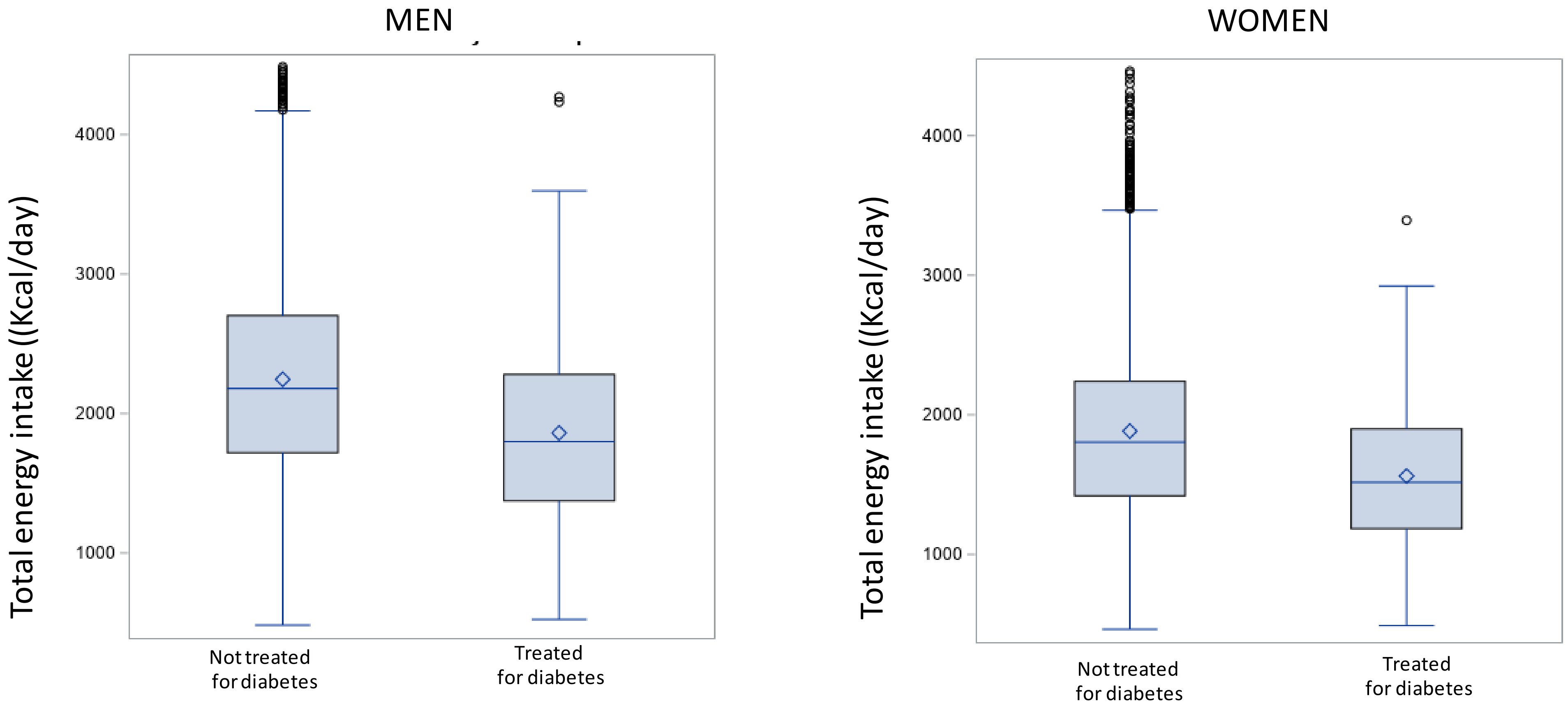

Supplement: Supplementary file 1 [file nutrients-12-00923-s001.pdf]
